# Cancrum Oris—Baptisia

**Published:** 1884-01

**Authors:** Walter M. James

**Affiliations:** Philadelphia


### CLINICAL BUREAU.

---

### CANCRUM ORIS—BAPTISIA.

WALTER M. JAMES, M. D., PHILADELPHIA.

November 8th, was called to see a child five years old, suffering from sore mouth. The roof of the mouth and the tongue were covered with small ulcers. An abundance of saliva flowed from the mouth, the tongue was covered over with a thick white, exceedingly moist coat, the edges being red. Breath fetid. I gave Mercurius and waited five days. At the end of this time I could see no perceptible improvement. I therefore studied the case anew. In Hering's *Condensed Materia Medica*, under *Baptisia*, I found the following symptoms, all of which agreed with the patient's condition:

Putrid ulceration of buccal cavity with salivation.

Well-developed ulcers.

Gums loose, flabby, dark red, and fetid.

Fauces dark red; putrid ulcers; can swallow liquids only.

Tongue white, with red papillæ, the edges red and shining.

I gave Baptisia$^{1m}$, and in three days the whole trouble had disappeared.

